# The Hemoglobin‐to‐Red Blood Cell Distribution Width Ratio as a Novel Inflammatory Biomarker for Arterial Stiffness Assessment

**DOI:** 10.1111/jch.70171

**Published:** 2025-10-24

**Authors:** Chengjie Zhu, Vipin Kumar, Megumi Narisawa, Kai Meng, Yanglong Li, Xian Wu Cheng

**Affiliations:** ^1^ Department of Intensive Care Unit (ICU) Yanbian University Hospital Yanji Jilin P. R. China; ^2^ Jilin Provincial Key Laboratory of Stress and Cardiovascular Disease Yanbian University Yanji Jilin P. R. China; ^3^ Department of Cardiology and Hypertension Yanbian University Hospital Yanji Jilin P. R. China; ^4^ Department of Cardiology Nagoya University Graduate School of Medicine Nagoya Japan; ^5^ Department of Nursing Nursing College Yanbian University Yanji Jilin P. R. China; ^6^ Department of Radiology Yanbian University Hospital Yanji Jilin P. R. China

**Keywords:** arterial stiffness, biomarker, hemoglobin to red blood cell distribution, inflammation

**Abbreviations**
cfPWV: carotid‐femoral pulse wave velocity
D: distance
HRR: hemoglobin‐to‐red cell distribution width ratio
IL: interleukin
RBC: red blood cell
RDW: red cell distribution width
TNF: tumor necrosis factor

Arterial stiffness is characterized by a progressive loss of vascular elasticity that results from structural and functional changes in the arterial wall. Arterial stiffness is thus both a consequence of vascular aging and a predictor of future cardiovascular events, and it has been shown to be an independent predictor of major adverse cardiovascular events, e.g., myocardial infarction, stroke, and cardiovascular mortality [1]. The early assessment of arterial stiffness has emerged as a valuable approach for cardiovascular risk assessment, offering the potential to identify high‐risk individuals before the onset of cardiovascular disease (Figure 1). Although the carotid‐femoral pulse wave velocity (cfPWV) is recognized as the gold standard for assessing arterial stiffness, it is not routinely measured in clinical practice due to high costs, technical complexity, and the need for skilled operators that have limited its widespread adoption [1]. In contrast, the hemoglobin‐to‐red cell distribution width ratio (HRR), calculated as the ratio of the patient's hemoglobin concentration (g/L) to the red cell distribution width (RDW), is a novel inflammatory marker that reflects the prognostic contributions of both hemoglobin and the RDW. The HRR has shown significant prognostic value in several diseases, including coronary artery disease [2], peripheral arterial disease [3], heart failure, and chronic kidney disease [4], highlighting its potential value as an indicator of systemic inflammation. However, the relationship between the HRR and arterial stiffness is unclear.

**FIGURE 1:**
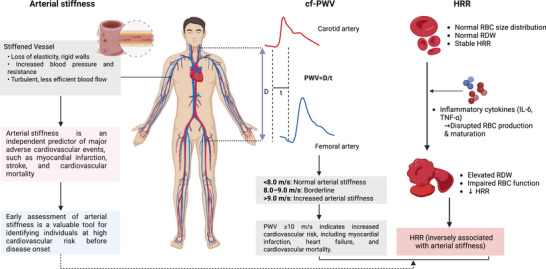
Assessment of arterial stiffness.

The study by Liu et al., in this issue of *The Journal of Clinical Hypertension*, presents compelling evidence for an inverse association between the HRR and arterial stiffness in a cohort of 3657 Chinese participants recruited between January 2016 and December 2023 [5]. The study addresses an important gap in cardiovascular risk assessment by examining whether commonly available hematological parameters could serve as biomarkers for vascular health. Liu et al. recruited participants from an initial cohort of 5886 subjects who underwent cfPWV measurement, with 2229 subjects excluded according to predefined criteria, yielding the final analytical sample. The comprehensive exclusion criteria appropriately eliminated individuals with acute cardiovascular events within the prior 6 months, congestive heart failure, severe arrhythmias, significant renal or hepatic dysfunction, malignancy, autoimmune disease, acute infectious disease, or current pregnancy and those using medications that could confound hematological parameters, such as folic acid and vitamin B12. The study's methodological strength is evident in its use of the cfPWV to measure arterial stiffness, a widely accepted and clinically relevant technique.

The measurements were performed with a Complior analyzer following standardized protocols, including calculating the recorded distance (D) as the measured D multiplied by 0.8. Arterial stiffness was defined as cfPWV ≥ 10 m/s. The participants were categorized into quartiles based on their HRR values: Q1 (HRR < 9.57), Q2 (> 9.57–< 10.55), Q3 (> 10.55–< 11.50), and Q4 (> 11.50). Their baseline characteristics revealed striking patterns across the HRR quartiles. Age decreased progressively from Q1 (64.1 ± 12.4 years) to Q4 (54.3 ± 11.7 years, *p* < 0.001), while the percentage of males increased markedly from 36.8% in Q1 to 88.5% in Q4. Importantly, the cfPWV values decreased consistently across quartiles: Q1 (10.2 ± 3.9 m/s), Q2 (9.6 ± 2.6 m/s), Q3 (9.6 ± 2.5 m/s), and Q4 (9.3 ± 2.2 m/s, *p* < 0.001). Correspondingly, the prevalence of arterial stiffness demonstrated a clear dose‐response pattern: Q1 (30.0%), Q2 (25.0%), Q3 (24.5%), and Q4 (20.5%) (*χ* ^2^ = 35.88, *p* < 0.001).

The Liu et al. study thus demonstrated a significant dose‐response relationship between HRR quartiles and arterial stiffness risk. In the unadjusted model, the odds ratios (ORs) for Q2, Q3, and Q4 were 0.93 (95% confidence interval [CI]: 0.89–0.97, *p* = 0.002), 0.92 (95%CI: 0.88–0.96, *p* < 0.001), and 0.88 (95%CI: 0.84–0.91, *p* < 0.001), respectively. In the fully adjusted model, the participants in the third quartile showed a 5% reduction in the odds of arterial stiffness (OR 0.95, 95%CI: 0.91–0.99, *p* = 0.024), while those in the highest quartile demonstrated a 7% reduction (OR 0.93, 95%CI: 0.88–0.97, *p* < 0.001) compared to the lowest quartile. Examining the HRR as a continuous variable revealed that each unit increase was associated with a 0.12 m/s decrease in the cfPWV (*β* = −0.12, 95%CI: −0.17–−0.06, *p* < 0.001) in the fully adjusted linear regression model. This linear relationship was confirmed by a restricted cubic splines analysis (*p* for non‐linearity = 0.277), strengthening the confidence in a true biological association rather than a statistical artifact. Additionally, the participants with arterial stiffness demonstrated significantly higher medication use, including antihypertensive (52.6% vs. 37.3%), antidiabetic (25.9% vs. 12.7%), and lipid‐lowering (36.2% vs. 27.7%) medications, compared to those without arterial stiffness (all *p* < 0.001). This suggests that the inverse association between the HRR and arterial stiffness persists despite intensive medical management.

The stratified analyses in Liu et al.’s study revealed a particularly valuable aspect of the investigation, showing stronger inverse associations in the participants with diabetes (OR 0.79, 95% CI: 0.67–0.94, *p* = 0.006) and hypertension (OR 0.84, 95% CI: 0.75–0.94, *p* = 0.002). These results suggest that inflammatory processes, as reflected by the HRR, are more important in individuals who are already experiencing vascular stress, supporting the biological plausibility of the proposed mechanisms. The significant interaction effects observed for both conditions (*p* for interaction = 0.031 for diabetes and 0.030 for hypertension) suggest that the HRR may be particularly relevant in high‐risk populations.

Liu et al. present a comprehensive and mechanistic discussion of how the HRR relates to arterial stiffness through two main pathways. First, increased RDW values and reduced hemoglobin levels reflect chronic inflammation, wherein pro‐inflammatory cytokines (e.g., interleukin‐6 and tumor necrosis factor [TNF]‐α) impair red blood cell (RBC) production and increase the cells' size variability while promoting vascular remodeling through abnormal collagen deposition and reduced elastin synthesis. Liu et al. strengthen this inflammatory hypothesis by citing research, including a meta‐analysis demonstrating cfPWV improvement following TNF‐α antagonist therapy and studies demonstrating positive associations between C‐reactive protein and arterial stiffness [6, 7]. Second, decreased hemoglobin and elevated RDW values may indicate impaired the body's erythrocyte antioxidant capacity. Reduced RBC deformability and increased cellular heterogeneity disrupt microvascular perfusion, promoting vascular oxidative stress through hemoglobin‐mediated nitric oxide quenching, iron‐catalyzed superoxide formation, and compromised antioxidant defenses. Liu et al. note that these inflammatory and oxidative stress pathways are amplified in diabetes and hypertension, which may explain why the HRR associations were stronger in these high‐risk populations.

Despite its strengths, the Liu et al. study has several limitations. The most significant of these is the cross‐sectional nature of the analysis. Although the study shows a strong relationship between the HRR and arterial stiffness, the authors cannot determine the cause or effect. It is unclear whether a low HRR contributes to the development of arterial stiffness, whether arterial stiffness and its associated inflammatory environment cause a reduction in the HRR, or whether both a low HRR and arterial stiffness are consequences of a shared underlying pathological process. Additionally, the study's single‐center design and recruitment of participants from a specific region in southern China limit the generalizability of the findings. Cardiovascular risk profiles and genetic backgrounds can vary significantly across different ethnic and geographic populations.

Another intriguing finding of that study is the inverse relationship between the HRR and conventional lipid parameters, e.g., total cholesterol and low‐density lipoprotein cholesterol, in the participants' baseline characteristics. This contradicts the typical positive association between dyslipidemia and cardiovascular risk. Liu et al. hypothesize that this may be due to the more prevalent use of lipid‐lowering medications among their participants with arterial stiffness. This highlights the complexity of interpreting observational data in treated populations.

In conclusion, Liu et al. provide valuable evidence supporting the HRR as a promising, inexpensive, and easily available biomarker for arterial stiffness. Their study is well‐executed, and the results are persuasive, particularly the stratified analyses highlighting the potential utility of the HRR in high‐risk groups such as diabetics and hypertensives. These findings reinforce the central role of inflammation and oxidative stress in vascular aging and provide a practical tool for clinicians to potentially identify individuals with subclinical vascular damage. Further studies are needed to establish the temporal relationship and determine whether the HRR can be used to predict the future development or progression of arterial stiffness. Additionally, interventional studies could explore whether improvements in the HRR, perhaps through anti‐inflammatory or antioxidant therapies, correlate with improvements in vascular health.

## Author Contributions

Chengjie Zhu wrote the first draft of the manuscript. Vipin Kumar and Megumi Narisawa drafted the figure. Yanglong Li and Kai Meng edited the manuscript. Xian Wu Cheng handled the funding and supervision.

## Conflicts of Interest

The authors declare no conflicts of interest.
